# Effects of Anthropogenic Disturbance of Natural Habitats on the Feeding Ecology of Moorish Geckos

**DOI:** 10.3390/ani13081413

**Published:** 2023-04-20

**Authors:** José Martín, Jesús Ortega, Roberto García-Roa, Gonzalo Rodríguez-Ruiz, Ana Pérez-Cembranos, Valentín Pérez-Mellado

**Affiliations:** 1 Departamento de Ecología Evolutiva, Museo Nacional de Ciencias Naturales, CSIC, C/José Gutiérrez Abascal 2, 28006 Madrid, Spain; 2 Ethology Lab, Cavanilles Institute of Biodiversity and Evolutionary Biology, University of Valencia, 46980 Valencia, Spain; 3 Departamento de Biología Animal, Universidad de Salamanca, 37007 Salamanca, Spain

**Keywords:** diet selection, feeding ecology, gecko, human disturbance, invertebrate prey, reptiles, *Tarentola*

## Abstract

**Simple Summary:**

Humans can alter the habitat quality and negatively affect many animals. However, some species seem able to cope with or even take advantage of these alterations. This study examines the effects of small anthropogenic alterations of a natural habitat on the feeding ecology of a gecko, *Tarentola mauritanica*. We compared geckos found in two contiguous small islands with either natural or human-altered seminatural habitats. Results showed that seminatural habitats differed from actual natural habitats in some microhabitat characteristics and in diversity of available prey. The diet of geckos also differed between habitats, being less diverse in altered habitats. However, the health state (body condition) of geckos was similar between habitats. These geckos seem able to modify their diet selection patterns to cope with anthropic disturbances of the habitat, which would allow them to inhabit and prosper in human-altered ecosystems.

**Abstract:**

Urbanization and anthropic influences can drastically modify a natural habitat and transform it into an easily recognizable “urban habitat”. Human activities can also induce less severe modifications of what apparently might still look like natural habitats. Therefore, these subtle alterations may be hidden but can still cause important negative effects on plant and animals. In contrast, some species seem able to take advantage of these anthropic alterations. Here, we examined the possible effects of the anthropogenic disturbance of an apparent natural habitat on the feeding ecology and body condition of Moorish geckos, *Tarentola mauritanica*. For this, we compared microhabitat structure, invertebrate availability, the diet composition (estimated from fecal contents), diet selection patterns and body condition of the two populations of geckos inhabiting two contiguous small islands. These islands have similar environmental characteristics, but highly contrasting differences in urbanization and anthropogenic influence. We found that, although the abundance of potential invertebrate prey was similar on both habitats, the diversity of invertebrate prey was lower in the altered habitat. As a consequence, although composition of the diet of geckos was similar on both islands, the diversity of prey and food niche breadth were lower in the altered habitat, and patterns of diet selection changed. However, these inter-habitat differences did not seem to affect the body size and body condition of geckos. We discuss how flexibility in feeding ecology may allow some species to cope with small anthropic disturbances of the habitat.

## 1. Introduction

Urbanization and other anthropic influences can cause severe drastic disturbances to the environment, transforming a natural habitat into an “urban habitat”, with peculiar and conspicuous differences that are immediately recognizable [1,2,3]. Human activities can also induce less severe, at times even subtle, modifications of a habitat that do not result in conspicuous changes in the “visual appearance” of that habitat. Consequently, these altered semi-natural habitats might still be mistaken for genuine natural ones, at least without doing detailed examinations of their state [4]. However, even some apparently minor and inconspicuous anthropogenic alteration of a habitat may be a threat to animals [2].

Although anthropic habitat alterations may cause the decline of many species [5], other species seem able to take advantage of the new resources and opportunities derived from anthropogenic disturbance, being even more abundant in human-altered habitats. For example, some species of lizards and geckos can be even more abundant in urban areas outside of their natural range [6,7]. The adaptation of animals to anthropic habitats is possible due to phenotypic changes derived from genomic adaptation after natural selection [8,9]. However, animals may also show behavioral flexibility [10,11] in, for example, microhabitat use and movements [12], habituation to inefficient human “predators” [13], or diet composition [14,15,16]. This behavioral plasticity might be especially important when animals inhabiting a natural habitat start to experience the effects of anthropic disturbances. Therefore, it is interesting to examine whether and how species that occupy habitats with different levels of anthropic influence can cope with habitat alterations and show behavioral flexibility.

The Moorish gecko, *Tarentola mauritanica*, is a Mediterranean species associated with rocky areas, where it can move easily thanks to the adhesive strips on its fingers [17]. In natural habitats, this gecko selects large rocks or groups of small rocks behind which to inhabit [18]. However, other populations of this gecko are anthropophilic, being found in human constructions (walls separating farms, buildings, cisterns, etc.) even inside cities, provided that there are shelters for them to hide behind [17,19]. The diet of *T. mauritanica* has been examined in different populations, as can be seen in review [17], showing that it is a generalist species that feeds mainly on ground-dwelling arthropods and other invertebrates, e.g., [19,20,21,22,23], but that its diet also includes some flying insects, especially inside urban areas [24]. However, it is not well known whether, in natural habitats that have suffered some anthropic disturbance, the expected effects on prey availability affect the diet composition of *T. mauritanica*. Moreover, it is not known whether this gecko is able to modify its diet selection patterns to respond to the presumed changes in available prey. Further, it is not known whether changes in diet derived from habitat alterations might directly affect the health state of geckos.

Here, we examined the possible effects of anthropogenic disturbances to and modifications of a natural habitat, which still might look natural in comparison with a truly unaltered natural habitat, on the feeding ecology of *T. mauritanica* geckos. For this, we compared two contiguous small islands with similar environmental characteristics (climate, vegetation, topography, etc.), but highly contrasting differences in urbanization and anthropogenic influence (actual unaltered natural habitats vs. apparently natural habitats with anthropic disturbances; see methods). In each island, we estimated the habitat structure, the availability of invertebrates in the habitat, the diet composition (based on fecal contents), diet selection patterns and body size and condition of geckos. We expect that the anthropogenic influence on the natural habitats should modify the habitat characteristics, which would affect the abundance or types of available prey and, therefore, the diet of geckos. We also expect that these modifications might have consequences on the body condition of geckos. However, geckos might be able to use flexibility to modify their diet selection patterns to cope with these human-induced changes.

## 2. Materials and Methods

### 2.1. Study Area

**Figure 1:**
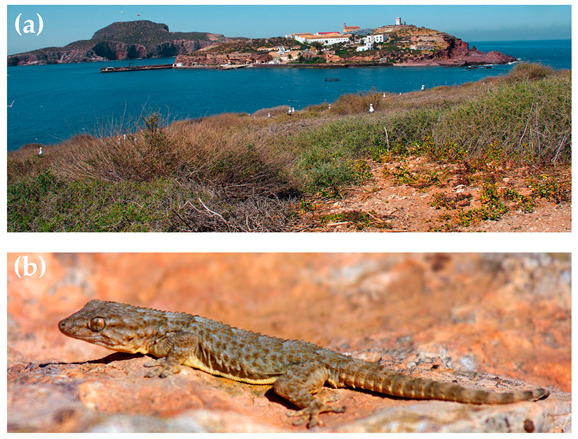
(**a**) Typical natural habitat at the Chafarinas Islands, with the uninhabited Rey Island in the foreground; the human-inhabited Isabel Island behind it in the middle, showing an apparently similar, but altered, seminatural habitat scattered between the buildings; and the Congreso Island in the background; (**b**) an adult *T. mauritanica* gecko on a rock in its natural habitat.

We performed the field work in the spring (March) of 2015 at the Chafarinas archipelago (Spain) (35°11′ N, 02°25′ W) (Figure 1a). These islands are located off the North African Moroccan coast in the western Mediterranean Sea, close to the shore, and are strictly protected as a nature reserve. The climate is a Mediterranean semiarid (dry and warm), the natural vegetation consists of woody bushes (Genus *Suaeda*, *Salsola* and *Lycium*) and there are abundant rocky areas where *T. mauritanica* geckos take refuge (Figure 1b). There are three islands, but abundant populations of *T. mauritanica* are found on only two of the islands (‘Isabel’ and ‘Rey’) [25]. One of the islands (‘Rey’; 13.9 ha) has been never been inhabited by humans and maintains highly restricted access to visitors (limited to occasional short visits by a few wardens and researchers); thus, this island maintains natural pristine conditions. Another island (‘Isabel’; 15.1 ha) has been inhabited by humans since ancient historical eras times. Nowadays, only a small human population (around 50 people) live there, but the island supported up to 5000 people at the beginning of the 20th century. Nearly 60% of its surface is covered with buildings or paved streets. However, close to and scattered between buildings, there are remains of seminatural vegetated areas. These areas are apparently similar to those found in the naturally preserved Rey Island, but they have been highly modified, as shown by the evidence of anthropogenic influence in some areas (soil erosion, organic and artificial residues, pathways marked with whitewashed rocks, presence of ornamental trees and bushes, etc.). Geckos are abundant in rocky areas in the natural areas; conversely, in seminatural areas, geckos are also abundant not only under rocks but also below anthropogenic materials, such as bricks or tiles. The third island (‘Congreso’) only holds several introduced individual geckos, which are restricted to a single uninhabited small concrete cabin used as a material store.

### 2.2. Effects of Human Disturbance on Microhabitat Characteristics

To characterize the microhabitats where we found *T. mauritanica* geckos on the two study islands (Isabel and Rey), we made a series of random transects covering all the available natural (on Rey Island) and seminatural (on Isabel island) habitats (i.e., excluding paved areas and buildings). Every 25 m, we chose the nearest rock to the transect point that might hold geckos (i.e., >10 cm length) as the center of a circular sampling area of 2 m diameter around the rock. In this area, we visually estimated percentage cover at the ground level of ‘bare-soil’ with gravel, ‘rocks’ (>10 cm) and ‘grasses’, and, above surface level, the cover of the dominant large woody ‘bushes’ and ‘mean bush height’ (Table 1). For similar methods, see [26]. One expected effect of human disturbance is an increase in soil compaction due to erosion and trampling, which might affect the abundance of soil invertebrates [2]. Hence, we measured ‘soil compaction’ with a hand penetrometer (Eijkelkamp Co., Em Giesbeek, The Netherlands) that we pushed slowly and vertically into the soil [27]. For each sampling point, we took and calculated the mean of five random measures of compaction from the area surrounding the sampling rock.

### 2.3. Availability of Potential Prey

We estimated the availability in the environment of invertebrates that could be potential prey of *T. mauritanica* geckos. In the same areas and microhabitats where we captured geckos, we randomly lifted rocks behind which geckos might take refuge (i.e., >10 cm length). Then, we counted the invertebrates (>2 mm long) for two minutes, identified to order or family level, that were found on the substrate below and on the undersurface of the turned rock, and in the50 cm radius around the rock. During the survey time, we counted invertebrates that were in this area or those which had escaped (e.g., isopods, centipedes or spiders), and also those that came flying and landed (flies, butterflies, etc.) (for similar procedures, see [28,29,30]). We also used a small stick to excavate the soil and leaf litter in the sampling area, looking for invertebrates buried close to the surface, such as insect larvae. With this standardized recording method, we estimated the relative abundance of the different types of invertebrates that were actually available in the gecko microhabitats.

### 2.4. Geckos Sampling Procedures

We conducted daily surveys to cover all the available natural and seminatural habitats on the two study islands (Isabel and Rey). We searched for *T. mauritanica* geckos by lifting rocks that active geckos used as refuges. To obtain diet samples, we collected the feces of live geckos captured by hand. We gently compressed the vents of geckos to force the expulsion of feces, and, sometimes, we had to place geckos in a plastic cage and wait some minutes until they voluntary expulsed the feces. Feces were stored individually in labeled Eppendorf vials. Sexes of adult individuals were identified by pressing gently around the cloacae to try the partial eversion of hemipenis of males. Juveniles could not be reliably sexed.

We used a metallic ruler to measure (to the nearest 1 mm) the ‘snout-to-vent length’ of geckos (SVL; i.e., the distance from the snout's tip to the posterior extreme of the cloacal scales) and ‘tail length’ and noted if the tail was original or regenerated. We measured ‘body mass’ with an electronic digital balance (0.1 g of precision). We calculated a ‘body condition index’ (BCI) using the residuals of the leastsquares linear regression between mass and total length (both log_10_-transformed) (*r* = 0.72, *F*_1,115_ = 126.95, *p* < 0.001). These residuals measure the condition of an animal independently of its body size [31,32]. We also calculated, as an alternative to the *BCI*, the ‘scaled mass index’ (SMI) [33] by following the formula: *SMI = Wi* [*L_0_*/*L_i_*]*^bSMA^*, where *W_i_* is the body mass and *L_i_* the *SVL* of a given individual, *L_0_* is the average *SVL* of all individuals, included as a standardized value to which compare each individual value, and *bSMA* is the slope of a standardized major axis regression. Body condition indices have been considered to constitute a way of estimating the health state of many animals, including reptiles [34,35].

To avoid sampling the same individuals twice, we did not sample the same areas twice. Geckos were released in good health a few minutes later at the exact point where they had been found.

### 2.5. Analyses of Fecal Contents of Geckos

We used a binocular microscope to identify to order or family level the prey remains present in fecal pellets. The analysis of fecal pellets is a useful non-invasive method to examine diet while avoiding damage to animals [28,36,37,38,39]. Only easily identified remains were used to conservatively estimate the numbers of each prey type per fecal pellet. To minimize missing soft-body prey that might have been destroyed by the digestive process [40], we carefully searched for the body parts of soft-body prey that were less likely to be digested (e.g., head capsules of insect larvae, chelicerae and cephalic region of spiders). In lizards, diet composition based on the visual analysis of fecal pellet contents is highly comparable to diet analyses based on gastric contents of killed animals [41], and results are even very close to those obtained for the molecular analyses (DNA metabarcoding) of invertebrate prey presence in fecal pellets [42].

### 2.6. Data Analyses

The availability in the habitat in each of the two islands of each class of invertebrate was characterized using the ‘abundance’ (total number) and ‘presence’ (percentage of sampling points where a particular type of invertebrate was found) values. Similarly, the diet of geckos was described using the ‘prey abundance’ (percentage of a given prey type in relation to the total number of prey) and the ‘prey presence’ (percentage of individual geckos that had consumed a given prey type) measures. Diversity of invertebrates in the habitat and in the diet were calculated using the Shannon–Weaver’s index, [43]: *H′ = −*Σ*p_i_ ln p_i_*, for the proportion (*p_i_*) of each of the taxonomic categories identified. To compare *H′* indices between islands, we used Hutcheson’s *t* test [44]. We also estimated the food niche breadth (*B*) of geckos using the inverse of Simpson’s diversity, index [45]: *B =* 1/Σ*p_i_^2^*, where *p_i_* is the proportion of prey resource *i*. This *B* index value was also calculated after standardizing it by dividing *B* by the number of actually used prey categories. To estimate the overlap in diet composition of geckos between islands, we used the symmetric index of Pianka [45]: *O_jk_ =* (Σ*p_ij_p_ik_*)/√(Σ*p_ij_^2^*)(Σ*p_ik_^2^*), where *p_ij_* is the relative occurrence of prey type *i* in the diet on the island *j* and *p_ik_* is the relative occurrence of prey type *i* in the diet on the island *k*. This index ranges from 0 to 1, with 1 indicating complete overlap.

The relationships between availability and diet within and between islands were first compared using Spearman’s rank correlations and *χ*^2^ tests. Then, in order to estimate selection for a prey type, we used the selectivity index (*D*) of Ivlev [46], modified by Jacobs [47], following the formula: *D =* (*r − p*)/(*r + p − 2rp*), where *r* is the proportion of a given prey type in geckos' diet and *p* is the proportion of that prey available in the environment. We selected this index because it is widely used in most studies of feeding preferences. However, the error in *D* may be high when there are low sample numbers in some prey categories [48]. To solve this problem, we calculated the relativized electivity index (E*) of Vanderploeg and Scavia [49]. This index is a measure of the feeder’s perception of a prey’s value, considering both its abundance and the abundance of other available prey types. The E* index follows the formula: E*_i_ = [*W_i_ −* (*1*/*n*)]/[*W_i_ +* (*1*/*n*)], which includes the number of prey types available (*n*) and the selectivity coefficient *W_i_ =* (*r_i_*/*p_i_*)/Σ*i*(*r_i_*/*p_i_*), which uses the proportions of prey *i* in the diet (*r_i_*) and in the environment (*p_i_*). These two selectivity indices range from −1 (total avoidance) through 0 (no or random selection) to +1 (maximum positive selection). We used *χ^2^* tests to test for the significance of electivities, comparing the observed proportions of each prey type in feces, with expected values being the proportions of prey available in the habitat (restricted to the types of prey actually consumed by geckos) [50].

## 3. Results

### 3.1. Effects of Human Disturbance on Microhabitat Characteristics

In spite of the fact that the visual “appearance” of the natural habitat might look similar on both islands, there were some significant differences between islands that could be related to the level of anthropogenic disturbance. Thus, on the island inhabited by humans (Isabel), there was a significantly lower coverage by bushes, but a significantly higher cover of grass, while the height of the bushes and the cover of bare soil and rocks did not significantly differ between islands. Additionally, soil compaction was significantly higher on the island with anthropogenic influence (Table 1). Moreover, considering only the data from Isabel Island, 56% of the seminatural sites sampled showed some signs of direct human disturbance, such as the presence of artificial residues (e.g., bricks, ceramics, glass, metals or plastic), whitewashed rocks, etc.

### 3.2. Availability of Potential Invertebrate Prey

We estimated the availability of invertebrates at 193 points, of which most sites (94.8%) contained some invertebrates larger than 2 mm (Table 2). Nevertheless, significantly more sites were empty of potential prey on Rey than on Isabel (8.1% vs. 1.2%; *χ*^2^ = 4.56, *p* = 0.033). Considering the rank-order importance of the different types of invertebrates, availability was similar on both islands (Spearman’s correlation, *r_s_* = 0.83, *n* = 18, *t* = 5.96, *p* < 0.0001). However, there were some differences in the relative proportions of the different types of invertebrates (*χ*^2^ = 57.79, df = 17, *p* < 0.0001) (Table 2). While on Rey the three most abundant invertebrate types were, in order of abundance, Formicidae (ants), Isopoda (isopods) and Coleoptera (beetles) (these three groups accounting for 69.1% of all invertebrates), on Isabel the most abundant types were Gastropoda (snails), Isopoda and Formicidae (76.1% of all invertebrates). The invertebrates most frequently found in the habitat on Rey were Gastropoda, Coleoptera and Isopoda, and, similarly but in a different order, on Isabel were Isopoda, Gastropoda and Coleoptera. The total number of available invertebrates was similar on both islands (one-way ANOVA, log-transformed data, *F*_1,191_ = 0.13, *p* = 0.71), but the diversity of invertebrate types was significantly higher on Rey (*H'* ± s^2^*_H_* = 1.93 ± 0.01) than on Isabel (1.80 ± 0.01) (Hutcheson’s *t* test, *t* = 2.53, df = 1670, *p* = 0.011).

### 3.3. Diet of the Geckos

We obtained a total of 117 fecal samples from *T. mauritanica* geckos (Table 3). The number of individual prey items that could be identified per fecal pellet ranged between 1 and 12 and was similar across islands (One-way ANOVA, log-transformed data, *F*_1,115_ = 0.79, *p* = 0.38) (Table 3). The composition of the diet of geckos was similar in rank order of importance between islands (Spearman’s correlation, *r_s_* = 0.78, *n* = 15, *t* = 5.22, *p* < 0.0001) and there was a high niche overlap between islands (*O* = 0.94). On both islands, the diet consisted mainly of Coleoptera, followed by Araneae and insect larvae, these being the most abundant and frequent prey types (these three types accounting for an overall 76% of prey), and other invertebrates were found in lower proportions (Table 3). However, there were some differences between islands in the relative contribution of the different prey types to the diet (*χ*^2^ = 37.29, df = 15, *p* < 0.005), and more different prey categories were consumed on Isabel (*n* = 15) than on Rey (*n* = 11). Moreover, the diversity of invertebrate types was significantly higher in the diet of geckos from Rey (*H'* ± s^2^*_H_* = 1.91 ± 0.01) than in geckos from Isabel (1.48 ± 0.01) (Hutcheson’s *t* test, *t* = 2.98, df = 229, *p* = 0.003). Similarly, food niche breadth of geckos was higher on Rey (*B* = 4.75; standardized *B* = 0.34) than on Isabel (*B* = 2.52; standardized *B* = 0.11).

### 3.4. Diet Selection Patterns

Overall, the diet of *T. mauritanica* geckos did not reflect the availability of invertebrates in the habitat (Spearman’s correlation, Rey: *r_s_* = -0.01, *n* = 18, *t* = −0.02, *p* = 0.99; Isabel: *r_s_* = 0.18, *n* = 18, *t* = 0.73, *p* = 0.47). Particularly, we may anecdotally state that some invertebrate types such as Gastropoda, Isopoda and Formicidae were not consumed in spite of being the most abundant invertebrates available in the habitat (Table 4). Neither Chilopoda, Thysanura nor Embiotera were consumed by geckos. Instead, geckos selected positively less abundant prey such as insect larvae or Dyctioptera. Additionally, Coleoptera and Araneae were selected on Isabel Island, where these varieties of prey were less abundant; conversely, on Rey, the higher availability of Coleoptera and Araneae seemed to fulfill the dietary requirements of geckos. Thus, they did not require a positive selection. Other invertebrates that were relatively scarce in the habitat, such as Lepidoptera, were also positively selected, while other minor types of prey were consumed in proportions similar to the expected by their availability in the habitat.

The diversity of invertebrates available in the habitat on Isabel was significantly higher than the diversity of prey in the diet of geckos from that island (Hutcheson’s *t* test, *t* = 2.72, df = 254, *p* < 0.007), whereas diversities of invertebrates in the habitat and diet were similar on Rey (*t* = 0.05, df = 101, *p* = 0.96).

### 3.5. Body Size and Body Condition of Geckos

There were not significant differences in the average body size of geckos between the islands (*SVL*, mean ± SE, Rey: 60.0 ± 1.0 mm, *n* = 39; Isabel: 60.0 ± 1.4 mm, *n* = 78; one-way ANOVA, log_10_-transformed, *F*_1,115_ = 0.27, *p* = 0.60). Considering the geckos that could be reliably sexed, which were mainly adults, there were not significant differences in *SVL* between sexes, although males tended to be larger (*SVL*, mean ± SE, males: 64.5 ± 1.5 mm, *n* = 44; females: 60.4 ± 1.3 mm, *n* = 39; one-way ANOVA, log_10_-transformed: *F*_1,81_ = 3.11, *p* = 0.08). Body mass depended strongly on tail condition and was not compared. 

Tail break rates did not significantly differ between islands (% of regenerated tails: Isabel = 65.4%, Rey = 61.5%; *χ*^2^ = 0.17, *p* = 0.68), but significantly more females than males had regenerated tails (males = 52.3%, females = 74.3%; *χ*^2^ = 4.31, *p* = 0.037). Sex ratios were significantly different between islands, with females being more abundant than males on Rey, with the inverse occurring on Isabel (sex ratio, Rey: 11 males:18 females = 0.61; Isabel: 33 males:21 females = 1.57; *χ*^2^ = 4.07, *p* = 0.044).

The body condition index (*BCI*) of geckos with their original tails was significantly lower than that of geckos with fully grown regenerated tails (which, although they were shorter, were much thicker at the basis) (*BCI*, mean ± SE, entire tail: −0.130 ± 0.023, *n* = 42, regenerated tail: +0.073 ± 0.021, *n* = 75; two-way ANOVA: *F*_1,113_ = 31.84, *p* < 0.0001), but there were no significant differences between the islands (Rey: +0.002 ± 0.025, *n* = 39; Isabel: −0.001 ± 0.024, *n* = 78; *F*_1,113_ = 0.18, *p* = 0.67) and the interaction was not significant (*F*_1,113_ = 0.15, *p* = 0.69).

Similarly, the scale mass index (*SMI*) did not significantly differ between islands (*SMI*, mean ± SE, Rey: 4.65 ± 0.10, *n* = 39; Isabel: 4.54 ± 0.11, *n* = 78; two-way ANOVA: *F*_1,113_ = 0.10, *p* = 0.75), nor did it depend significantly on the tail state (entire tail: 4.46 ± 0.11, *n* = 42, regenerated tail: 4.64 ± 0.11, *n* = 75; *F*_1,113_ = 2.12, *p* = 0.15). Additionally, the interaction was not significant (*F*_1,113_ = 1.88, *p* = 0.17).

## 4. Discussion

We found some differences in microhabitat characteristics between islands. They likely resulted from anthropic disturbance and could further explain the differences observed in diversity, but not in abundance, of invertebrates available in those habitats. These differences in prey availability were reflected in the differences of the diversity of prey consumed by *T. mauritanica* geckos. However, geckos seemed to respond with flexibility, modifying the patterns of prey selection to maintain their diet composition. This probably allowed geckos to maintain their body condition, regardless of anthropic habitat alterations.

The patches of seminatural habitats that remained, scattered between buildings, on the human-inhabited island (Isabel) were apparently similar to the natural habitats of the uninhabited island (Rey) and held high densities of *T. mauritanica* geckos and other species of reptiles [25]. However, a detailed examination of microhabitat structure revealed that seminatural habitats had suffered a clearing of bushes, which had been replaced by more extended grassy areas. Additionally, the substrate was more compact, which was likely due to an effect of erosion and trampling. Nevertheless, the proportion of rocks that geckos could use as a refuge remained similar. Moreover, on the island with human influence, geckos were also found under artificial rests, such as bricks or roof tiles, which also provided cover and may have even favored the apparent high abundance of *T. mauritanica* in this island. In fact, the use of artificial covers provided by researchers had been used as a management measure for other species of saxicolous lizards and geckos [51]. 

These changes in microhabitat structure, in likely combination with other indeterminate human influences, can very likely explain the changes observed in the available invertebrates. Although the total abundance of invertebrates did not change between natural and seminatural habitats, there were differences in the abundances of each invertebrate type. Some invertebrate types seemed to be favored by human disturbance and were more abundant in seminatural habitats (e.g., isopods and gastropods), while other decreased their numbers (e.g., beetles and ants), leading to low values of diversity, and probably a loss of species. This is an expected effect of human disturbance. Indeed, many studies have highlighted that human global alterations are leading to a mass loss of many invertebrates and identified the drivers of this decline [52]. Human alterations may also favor that pest insect populations flourish in urban areas [53]. Nevertheless, for a generalist predator, such as *T. mauritanica* geckos, the loss of diversity and complexity of the invertebrate fauna might not be important if the few remaining species, or the new pest ones, maintain high numbers and are still appropriate as prey. 

The diet composition of *T. mauritanica* on these islands was similar to those observed in other Mediterranean populations [19,20,21,22,23,24], including some ground-dwelling arthropods (mainly beetles, spiders and larvae) but also flying ones (especially on the uninhabited island). Interestingly, considering the main prey types, there were not substantial differences between islands, which showed a very high overlap in diet composition. Hence, it seemed that geckos were able to maintain their diet in spite of habitat anthropic alterations, at least during the favorable spring season. However, seasonal changes in invertebrate availability, such as the drastic decreases in abundance and diversity due to the summer drought periods [54], potentially had a greater impact on the altered habitats, impeding geckos from attaining their dietary requirements. Moreover, even in spring, the diversity of prey types in the diet and the food niche breadth were already lower on the inhabited island with altered habitats. Similarly, a previous study also showed that the diet of *T. mauritanica* was more varied in rural areas than in cities [55]. Additionally, rock agamas from urban areas, namely *Psammophilus dorsalis*, hada broader range of prey items as they magnified their myrmecophagous specialization in these urban habitats [56]. The question that arises is whether a less diverse diet has the same nutritional value. Studies with captive reptiles have shown that a more diverse diet ensures the ingestion of a broader range of nutrients and limits the likelihood of nutritional disease occurrence [57].

The diet of *T. mauritanica* did not directly reflect the availability of invertebrates in the habitat in any of the islands. Rather, geckos consistently selected some prey types, while they avoided other invertebrates regardless of their abundance. Therefore, *T. mauritanica* geckos showed clear food preferences, similar to those described in other populations [23]. Geckos might try to maintain these preferences on both islands, regardless of the state of the habitat and the available prey. However, this could be a problem because inhabiting the altered habitat, even for some preferred prey types (beetles and spiders), requires that geckos make a stronger positive selection (i.e., probably a higher foraging effort). In contrast, in the natural habitat, the relative availability of the preferred prey types is more similar to their presence in the diet. Additionally, we cannot rule the possibility that the preference of Isabel’s geckos for some prey types that are no longer abundant might be due not only to habitat alterations but also to competition for the same food item by other species (lizards, etc.) that might be favored by the anthropic influence and be more abundant here [58,59].

Moreover, the diversities of prey available and consumed were similar in the unaltered habitat. Conversely, in the altered habitat, the diversity of prey consumed was lower than expected in relation to the availability of prey. Therefore, feeding requirements of *T. mauritanica* geckos were constrained in some way in the altered habitat, as geckos maintained their feeding preferences for prey that were less abundant, but avoided some of the most abundant invertebrates found there. Other anthropophilic animal species were, however, more able to exploit anthropogenic food sources, even those new food items that are not found in natural diets but instead thrive in urban environments [10,14,16]. Nevertheless, it might be possible that our study population of *T. mauritanica* of seminatural areas had not yet entirely adapted to human disturbance, while “pure” urban populations might have shown greater flexibility in feeding behavior.

In spite of all the potentially negative effects of anthropic alteration of the habitat, *T. mauritanica* geckos seemed able to maintain a good health state, as suggested by their similar body condition values on both islands. Human disturbance has been shown to affect body condition in other reptiles. For example, deforestation affects tropical forest geckos, *Cyrtodactylus* spp., with geckos having lower body condition in patches with low canopy cover, which, although not examined, might be attributed to lower prey availability [60]. However, in rock lizards, *Iberolacerta cyreni*, the loss of body condition in altered habitats was mainly explained by the increased use of costly behavioral strategies to cope with the increased predation risk due to the human-induced loss of natural refuges [61]. In green anole lizards *Anolis carolinensis*, the level of human habitat modification was correlated with body condition of lizards, although only in females; however, this effect did not seem to be directly related to changes in arthropod abundance or biomass [15]. Additionally, in another study of *T. mauritanica* geckos, the lower body condition of geckos observed in urban habitats, in comparison with rural areas, was explained as a consequence of pollution by heavy metals, rather than by changes in diet [55]. In contrast, rural rock agamas, *P. dorsalis*, had lower body conditions than urban lizards, despite the greater diversity of prey types and the larger volume of food consumed [56]. This was explained by the higher movement rates, with presumed associated higher energy expenditure, of lizards in rural areas. Nevertheless, it has been suggested that in many species the body condition might be poorly correlated with body lipid reserves, and that these levels of reserves would not always be directly related to fitness [62]. Thus, it is likely that, in order to examine the actual effects of human alterations on the health state of *T. mauritanica* geckos, it will be necessary to consider several alternative physiological parameters (i.e., immune response, stress levels, etc.) [34].

## 5. Conclusions

The small effects of human disturbances of natural habitats may induce alterations of a habitat and its fauna that may be not so apparent at first sight, but that require examination. This is important in a changing world scenario, because otherwise many threats to biodiversity may be easily overlooked. In our study area, included in a nature reserve, we found that these seminatural habitats may have significant alterations in the microhabitat structure that likely, or at least partly, explain the changes in the invertebrate fauna. Further, these changes should likely affect the diet of insectivorous predators, such as that of the Moorish gecko, *T. mauritanica*. However, in spite of these changes, it seems that this gecko species is able to cope with the potential negative effects of human alterations and maintain abundant populations with similar body conditions to those possessed by geckos from natural habitats. This flexibility in responses to human disturbances may allow some species, such as this variety of gecko, to proliferate in human-transformed habitats. However, it remains to be examined in greater detail how the different effects and different levels of human alterations may affect the actual physiological health state of apparently "anthropophilic" animals. This would allow researchers to make predictions and take actions to minimize potential future conservation problems.

## Figures and Tables

**Table 1:** Characteristics of microhabitats (mean ± SE) at two of the Chafarinas Islands with different levels of anthropogenic disturbance (natural: Rey; altered: Isabel) based on 54 random habitat samples. The statistics (*F* and *p*) are given from one-way ANOVAs tests comparing both islands. Significant comparisons are marked in bold.

	Rey Island (Natural) (*n* = 29 Points)	Isabel Island (Altered) (*n* = 25 Points)		
	Mean ± SE	Mean ± SE	*F*_1,52_	*p*
Bare soil with gravel (%)	55.0 ± 4.8	42.8 ± 4.2	3.52	0.066
Rocks (%)	27.1 ± 3.8	23.8 ± 3.1	0.43	0.52
Grasses (%)	17.9 ± 3.4	33.4 ± 4.5	7.89	**0.007**
Bushes (%)	27.2 ± 4.5	15.0 ± 2.4	5.21	**0.027**
Mean bush height (cm)	52 ± 9	74 ± 12	2.30	0.13
Soil compaction (kg cm^−2^)	1.11 ± 0.13	1.71 ± 0.19	6.84	**0.011**

**Table 2:** Total abundance of invertebrates (>2 mm) available at two of the Chafarinas Islands with different levels of anthropogenic disturbance (natural: Rey; altered: Isabel) based on 193 random habitat samples. ‘Abundance’ (total number, % and mean number ± SE of organisms at each sampling point) and ‘Presence’ (proportion of sites containing a particular organism) are given.

	Rey Island (Natural) *n* = 111 Points				Isabel Island (Altered) *n* = 82 Points			
	Abundance			Presence	Abundance			Presence
	*n*	%	Mean ± SE	%	*n*	%	Mean ± SE	%
Gastropoda	112	9.9	1.01 ± 0.15	37.8	172	21.9	2.10± 0.36	63.4
Pseudoscorpion	4	0.3	0.04 ± 0.02	8.0	6	0.8	0.07 ± 0.05	3.7
Araneae	36	3.2	0.32 ± 0.07	23.4	18	2.3	0.22 ± 0.06	18.3
Opiliones	2	0.2	0.02 ± 0.01	1.8	1	0.1	0.01 ± 0.01	1.2
Acarina	3	0.3	0.03 ± 0.03	0.9	13	1.6	0.16 ± 0.07	8.5
Isopoda	198	17.5	1.78 ± 0.44	31.5	283	36.0	3.45 ± 0.59	54.9
Chilopoda	41	3.6	0.37 ± 0.08	23.4	15	1.9	0.18 ± 0.07	9.8
Thysanura	84	7.4	0.76 ± 0.19	25.2	26	3.3	0.32 ± 0.15	12.2
Dictyoptera	5	0.4	0.02 ± 0.01	3.6	3	0.4	0.04 ± 0.02	3.7
Embioptera	27	2.4	0.02 ± 0.01	5.4	1	0.1	0.01 ± 0.01	1.2
Homoptera	2	0.2	0.02 ± 0.01	1.8	1	0.1	0.01 ± 0.01	1.2
Heteroptera	11	1.0	0.10 ± 0.08	2.7	3	0.4	0.04 ± 0.03	2.4
Diptera	11	1.0	0.10 ± 0.03	9.9	6	0.8	0.07 ± 0.04	4.9
Lepidoptera	3	0.3	0.03 ± 0.02	2.7	2	0.3	0.02 ± 0.02	2.4
Coleoptera	173	15.3	1.56 ± 0.46	25.9	85	10.8	1.04 ± 0.17	48.8
Hymenoptera	2	0.2	0.02 ± 0.01	1.8	2	0.2	0.02 ± 0.02	2.4
Formicidae	409	36.3	3.68 ± 9.91	22.5	144	18.3	1.76 ± 0.56	39.0
Insect larvae	5	0.4	0.05 ± 0.02	3.6	6	0.8	0.07 ± 0.03	7.9
								
Total Invertebr.	1128	100	10.16 ± 1.08	91.9	787	100	9.60 ± 1.02	98.8

**Table 3:** Composition of the diet of the Moorish gecko, *Tarentola mauritanica*, in two of the Chafarinas Islands with different levels of anthropogenic disturbance (natural: Rey; altered: Isabel), based on 117 fecal samples collected from live geckos. ‘Abundance’ (total number, % and mean number ± SE of prey in each fecal sample) and ‘Presence’ (percentage of fecal samples containing a particular prey item) are shown.

	Rey Island (Natural) *n* = 39 Fecal Pellets				Isabel Island (Altered) *n* = 78 Fecal Pellets			
	Abundance			Presence	Abundance			Presence
	*n*	%	Mean ± SE	%	*n*	%	Mean ± SE	%
Gastropoda					3	1.5	0.04 ± 0.02	3.8
Pseudoscorpion	3	3.5	0.08 ± 0.04	7.7	2	1.0	0.03 ± 0.02	2.6
Araneae	11	12.8	0.28 ± 0.07	28.2	23	11.7	0.29 ± 0.06	26.9
Opiliones					1	0.5	0.01 ± 0.01	1.3
Isopoda	2	2.3	0.06 ± 0.04	5.1	1	0.5	0.01 ± 0.01	1.3
Dictyoptera	9	10.5	0.23 ± 0.09	17.9	6	3.0	0.08 ± 0.03	7.7
Homoptera	2	2.3	0.05 ± 0.04	5.1	1	0.5	0.01 ± 0.01	1.3
Heteroptera	2	2.3	0.05 ± 0.04	5.1	1	0.5	0.01 ± 0.01	1.3
Diptera	4	4.5	0.10 ± 0.05	10.3	5	2.5	0.06 ± 0.03	6.4
Lepidoptera	9	10.5	0.23 ± 0.07	23.1	5	2.5	0.06 ± 0.03	6.4
Coleoptera	34	39.5	0.87 ± 0.18	51.3	120	60.9	1.56 ± 0.25	60.3
Hymenoptera					1	0.5	0.01 ± 0.01	1.3
Formicidae					2	1.0	0.03 ± 0.02	2.6
Insect larvae	9	10.5	0.23 ± 0.07	23.1	18	9.1	0.23 ± 0.06	21.8
Arthropoda indet.	1	1.2	0.03 ± 0.03	2.6	8	4.1	0.10 ± 0.04	10.3
								
Total prey	86	100	2.21 ± 0.20	100	197	100	2.53 ± 0.25	100

**Table 4:** Prey selection by the Moorish gecko, *Tarentola mauritanica*, in two of the Chafarinas Islands with different levels of anthropogenic disturbance (natural: Rey; altered: Isabel). The electivity index of Jacobs (*D*) and the Vanderploeg and Scavia’s relativized electivity index (E*) are given for each potential prey type. The statistical significance (*p* from a *χ^2^* test) of this E* index is given. Significant electivities are marked in bold.

	Rey Island (Natural)			Isabel Island (Altered)		
	Electivity Index			Electivity Index		
	*D*	E*	*p*	*D*	E*	*p*
Gastropoda	−1	−1		**−0.895**	**−0.957**	**<0.0001**
Pseudoscorpion	+0.821	+0.514	0.058	+0.143	−0.407	0.79
Araneae	+0.633	+0.119	0.20	**+0.699**	**+0.236**	**<0.0001**
Opiliones	−1	−1		+0.601	+0.117	0.32
Acarina	−1	−1		−1	−1	
Isopoda	**−0.799**	**−0.919**	**<0.0001**	**−0.982**	**−0.991**	**<0.0001**
Chilopoda	−1	−1		−1	−1	
Thysanura	−1	−1		−1	−1	
Dictyoptera	**+0.927**	**+0.764**	**<0.0001**	**+0.783**	**+0.434**	**0.001**
Embioptera	−1	−1		−1	−1	
Homoptera	+0.861	+0.612	0.068	+0.601	+0.117	0.32
Heteroptera	+0.416	−0.139	0.34	+0.143	−0.407	0.86
Diptera	+0.664	+0.203	0.28	+0.544	+0.027	0.052
Lepidoptera	**+0.956**	**+0.851**	**<0.0001**	**+0.822**	**+0.520**	**0.0013**
Coleoptera	+0.566	−0.101	0.91	**+0.859**	**+0.282**	**<0.0001**
Hymenoptera	−1	−1		+0.334	−0.225	0.61
Formicidae	−1	−1		**−0.912**	**−0.965**	**<0.0001**
Insect larvae	**+0.927**	**+0.764**	**<0.0001**	**+0.858**	**+0.583**	**<0.0001**

## Data Availability

The data presented in this study are openly available in FigShare at https://doi.org/10.6084/m9.figshare.22296838.
